# Supplementary material for: Immunomodulatory Effect of Human Lactoferrin on Toll-like Receptors 2 Expression as Therapeutic Approach for Keratoconus
Source: Int J Mol Sci. 2022 Oct 15;23(20):12350. doi: 10.3390/ijms232012350 (PMC9604127; doi:10.3390/ijms232012350)
Supplement: Supplementary file 1 [file ijms-23-12350-s001.zip › ijms-1929584-supplementary.pdf]

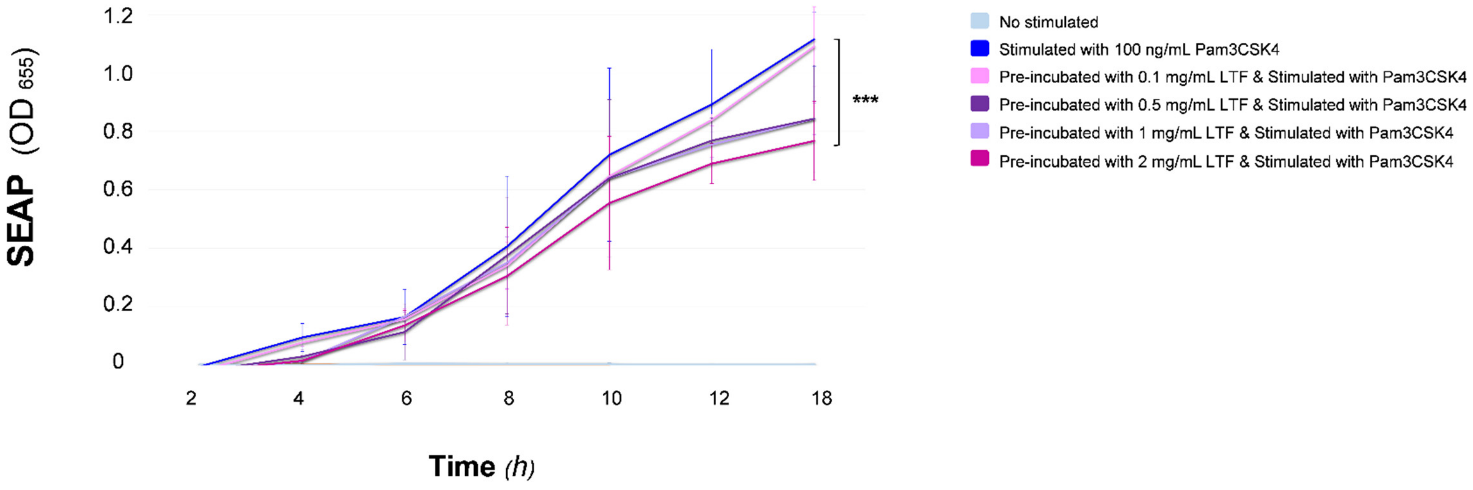

Figure S1. LTF dose test. Statistical differences: ***p<0.0001 between cells stimulated with 100ng/mL Pam3CSK4 and cells pre-incubated with 2mg/mL LTF. Abbreviations: LTF, lactoferrin; Pam3, Pam3CSK4; SEAP, secreted embryonic alkaline phosphatase.

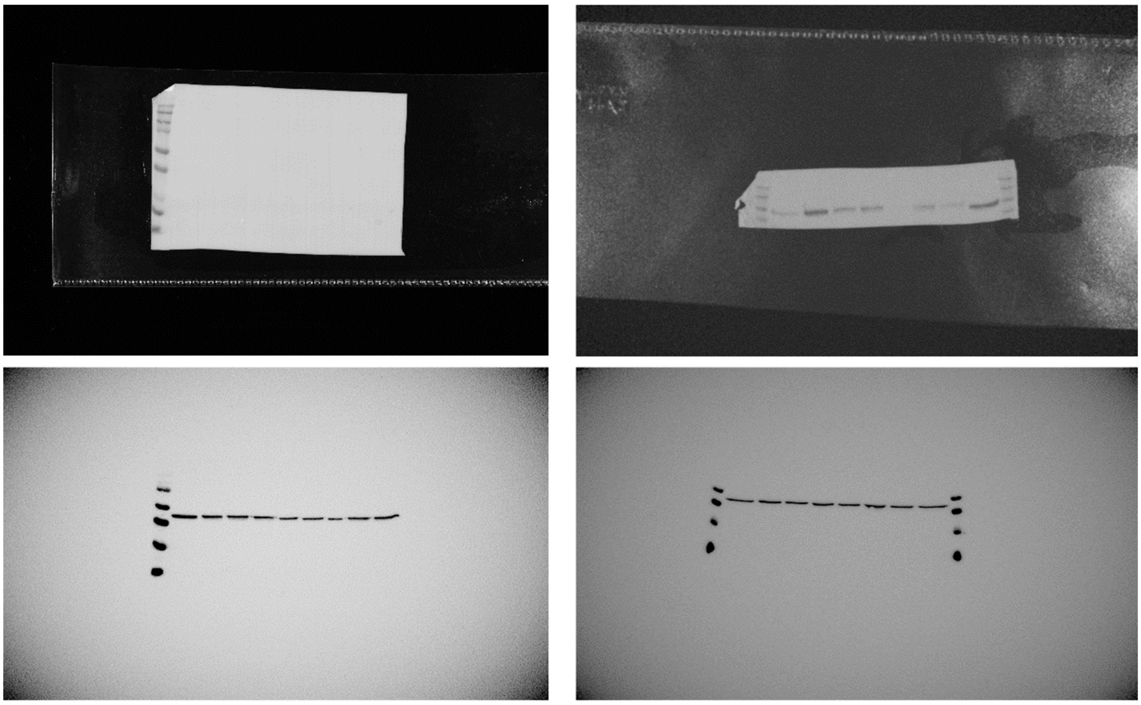

Figure S2. Uncropped blots in the same order as in Figure 4C.
